# Liver Metastases in Pancreatic Acinar Cell Carcinoma Treated with Selective Internal Radiation Therapy with Y-90 Resin Microspheres

**DOI:** 10.1155/2017/1847428

**Published:** 2017-10-11

**Authors:** Felipe Nasser, Joaquim Maurício Motta Leal Filho, Breno Boueri Affonso, Francisco Leonardo Galastri, Rafael Noronha Cavalcante, Diego Lima Nava Martins, Vanderlei Segatelli, Lilian Yuri Itaya Yamaga, Rene Claudio Gansl, Bernardino Tranchesi Junior, Antônio Luiz de Vasconcellos Macedo

**Affiliations:** ^1^Interventional Radiology Unit, Hospital Israelita Albert Einstein, Av. Albert Einstein 627/701, Morumbi, 05652-900 São Paulo, SP, Brazil; ^2^Pathology Department, Hospital Israelita Albert Einstein, Av. Albert Einstein 627/701, Morumbi, 05652-900 São Paulo, SP, Brazil; ^3^Nuclear Medicine Unit, Hospital Israelita Albert Einstein, Av. Albert Einstein 627/701, Morumbi, 05652-900 São Paulo, SP, Brazil; ^4^Oncology Department, Hospital Israelita Albert Einstein, Av. Albert Einstein 627/701, Morumbi, 05652-900 São Paulo, SP, Brazil; ^5^Cardiology Department, Hospital Israelita Albert Einstein, Av. Albert Einstein 627/701, Morumbi, 05652-900 São Paulo, SP, Brazil; ^6^General and Oncological Surgery, Hospital Israelita Albert Einstein, Av. Albert Einstein 627/701, Morumbi, 05652-900 São Paulo, SP, Brazil

## Abstract

**Background:**

Pancreatic acinar cell carcinoma (PACC) is a rare tumor. Surgical resection is the treatment of choice when feasible, but there are no clear recommendations for patients with advanced disease. Liver-directed therapy with Y-90 selective internal radiation therapy (SIRT) has been used to treat hepatic metastases from pancreatic tumors. We describe a case of PACC liver metastases treated with SIRT.

**Case Report:**

59-year-old man was admitted with an infiltrative, solid lesion in pancreatic tail diagnosed as PACC. Lymph nodes in the hepatic hilum were enlarged, and many metastatic liver nodules were observed. After partial pancreatectomy, the left and right lobes of the liver were separately treated with Y-90 resin microspheres. Follow-up imaging revealed that all hepatic nodules shrank by at least 50%, and 3 nodules disappeared completely. Lipase concentration was 8407 U/L at baseline, rose to 12,705 U/L after pancreatectomy, and declined to 344 U/L after SIRT. Multiple rounds of chemotherapy in the subsequent year shrank the hepatic tumors further; disease then progressed, but a third line of chemotherapy shrank the tumors again, 16 months after SIRT treatment.

**Conclusion:**

SIRT had a positive effect on liver metastases from PACC. In conjunction with systemic therapy, SIRT can achieve sustained disease control.

## 1. Introduction

Pancreatic acinar cell carcinoma (PACC) is a rare tumor that accounts for approximately 1% of malignant pancreatic neoplasms [1]. Surgery is the treatment of choice in these patients, particularly for early-stage disease. Chemotherapy and radiotherapy have been used in locally advanced or metastatic disease, but their efficacy has not been studied in controlled, prospective studies, and there are no definitive guidelines for treating advanced PACC [2–4].

Selective internal radiation therapy (SIRT) with yttrium-90 (Y-90) resin microspheres is an alternative treatment for patients with primary or secondary liver malignancies not amenable to resection [5, 6]. During SIRT, radiotherapy is delivered directly to the liver by superselective intra-arterial catheterization. Several studies have reported the safety and efficacy of SIRT in treating hepatocellular carcinoma, metastatic colorectal cancers, and neuroendocrine tumors [7–9]. SIRT may also benefit patients with other primary or secondary liver tumors, such as cholangiocarcinoma; sarcoma; and metastases from breast, cervical, pancreatic, and lung cancers [10].

In the context of pancreatic cancer, SIRT has been used as a salvage therapy [11, 12] or in combination with systemic therapy [13] to treat hepatic metastases from pancreatic exocrine tumors and neuroendocrine tumors [14, 15]. There are few cases reported in the literature, most of them using SIRT to treat pancreatic adenocarcinoma liver metastases. The response rate (complete or partial response according to mRECIST) described is around 40% and the median overall survival is around 9 months after SIRT [16]. Here, we describe the case of a patient with liver metastases from PACC treated with SIRT. This study was approved by the Institutional Review Board of our Hospital.

## 2. Case Presentation

A 59-year-old man underwent a transabdominal ultrasound to investigate persistent postprandial abdominal pain and was admitted to the hospital with liver nodules of unknown cause. Most laboratory values were close to normal, including the tumor markers alpha-fetoprotein, carcinoembryonic antigen, and carbohydrate antigen 19-9; however, serum lipase concentrations were elevated (Table 1).

**Figure 1:**
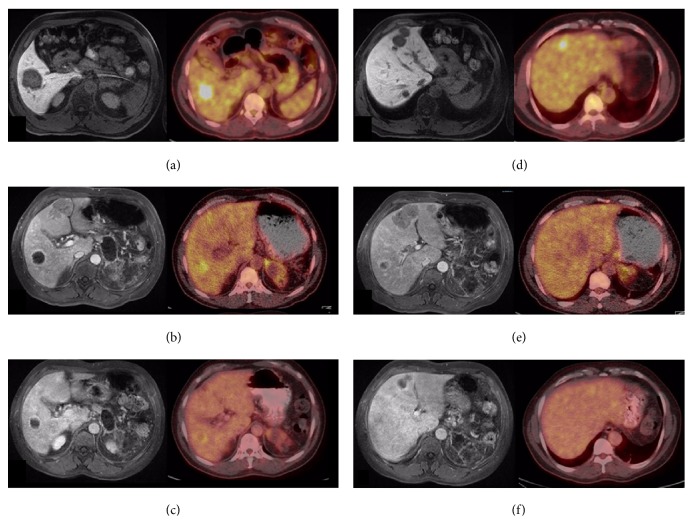
Magnetic resonance (MR) and positron emission tomography-computed tomography with 18-fluorodeoxyglucose (18-FDG PET-CT) imaging. (a–c) Axial MR images in the portal phase (left column) and axial 18-FDG PET-CT images (right column) showing the evolution of the largest hypovascular and hypermetabolic liver nodule in the* right lobe*: (a) at admission, (b) 30 days after the first SIRT session, and (c) 45 days after the second SIRT session. (d–f) Axial MR images in the portal phase (left column) and axial 18-FDG PET-CT (right column) showing evolution of the largest hypovascular and hypermetabolic liver nodules in the* left lobe*: (d) at admission, (e) 30 days after the first SIRT session (note that the lesions had grown), and (f) 45 days after the second SIRT session.

An abdominal, contrast-enhanced computed tomography (CT) scan and magnetic resonance imaging (MRI) showed multiple, unresectable hypovascular liver nodules in both lobes (Figures 1(a) and 1(d)). The largest, in segment V, was 5.3 cm in diameter (Table 2). The scans also showed an infiltrating, poorly delimited, solid lesion in the pancreatic tail that was invading and causing thrombosis of the splenic vein. Lymph nodes in the hepatic hilum were enlarged.

Positron emission tomography- (PET-) CT with 18-fluorodeoxyglucose (18-FDG) showed that the pancreatic and hepatic lesions were hypermetabolic, with a maximum standardized uptake value of 8.5. An ultrasound-guided biopsy of the largest hepatic nodule was performed on the same day as the PET-CT, and the histopathologic analysis was suspicious for PACC. Whole-body PET-CT with a somatostatin analog (^68^Ga-DOTATATE) revealed no lesion suggestive of a tumor highly expressing somatostatin receptors, which excluded a diagnosis of well-differentiated neuroendocrine tumors. No extrahepatic metastases were evident.

**Figure 2:**
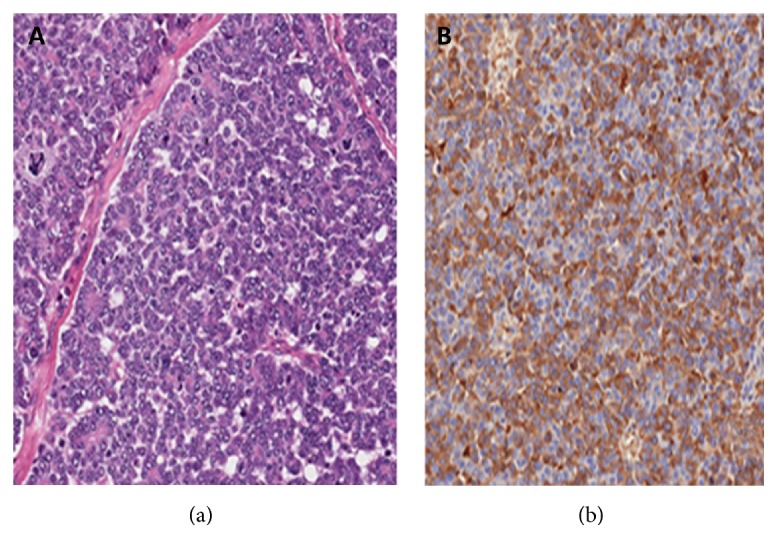
Histopathology of the resected pancreas. (a) Neoplasm with a solid acinar architectural microscopic pattern (hematoxylin and eosin, 20x); (b) positive immunostaining (brown; counterstained with hematoxylin and eosin) for alpha-1-antitrypsin in neoplastic cells.

The tumor board (composed of a clinical oncologist, oncological surgeon, and interventional radiologist) decided to resect the primary tumor and treat the liver metastases with SIRT. Systemic chemotherapy was contraindicated because the patient was living abroad and would not be able to attend follow-up appointments in our country. Pathologic (microscopic examination) analysis of the resected pancreatic body and tail showed that the tumor was characterized by marked cellularity and a paucity of fibrous stroma. The neoplastic cells were arranged in solid nests and in some areas formed an acinar arrangement (Figure 2(a)), with round or oval nuclei, moderate pleomorphism, prominent nucleoli, and eosinophilic granular cytoplasm. The neoplastic cells were focally positive for periodic acid-Schiff (PAS) stain and resistant to diastase digestion. In an immunohistochemical study, the tumor cells were diffusely immunoreactive for CK18 and focally positive for CK7, alpha-1-antitrypsin (Figure 2(b)), and alpha-1-antichymotrypsin. Scattered cells were positive for synaptophysin and chromogranin A. The neoplastic cells were not immunopositive for alpha-fetoprotein.

The patient then underwent SIRT to control the secondary liver lesions. Because of the extent of the liver lesions, SIRT was performed in 2 stages. First, Y-90 resin microspheres (20 to 60 *μ*m, SIR-Spheres; Sirtex Medical Limited, North Sydney, Australia) were delivered to the right hepatic lobe, and 1 month later microspheres were delivered to the left hepatic lobe and a right hepatic artery branch to segments IV/VIII.

**Figure 3:**
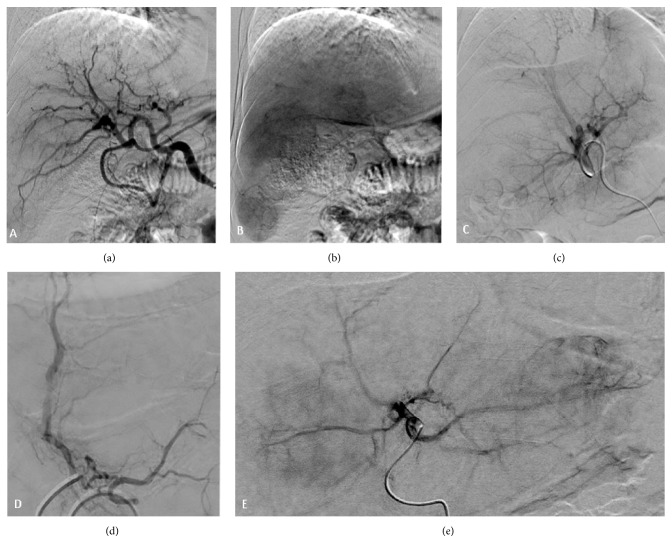
Digital subtraction angiography: (a) Angiogram showing the anatomy of the common hepatic artery. (b) Late phase showing some of the nodules. (c) Angiogram of the right hepatic artery before SIRT. (d) Catheterization of the arterial branch feeding segments IV/VIII. (e) Angiogram of the left hepatic artery before SIRT.

In the first SIRT treatment, a pulmonary shunt of 6.5% was detected. We used the Dose Activity Calculator (http://apps01.sirtex.com/smac/) [17, 18] to calculate the appropriate dose and administered a Y-90 dose of 1.36 GBq (37 mCi equivalent) through the right hepatic artery and a dose of 0.34 GBq through the artery branch for segment VIII (part of the nodule in segment IV was being fed by this branch) (Figures 3(a), 3(b), and 3(c)). A follow-up MRI 30 days after the first administration of SIRT showed that the nodules in the treated right lobe of the liver had shrunk, whereas the lesions in the untreated left lobe had grown (Figures 1(b) and 1(e), Table 2).

In the second SIRT treatment, a pulmonary shunt of 5% was detected, and a dose of 0.25 GBq (6.7 mCi) was delivered through the right hepatic artery branch to segments IV and VIII (to treat the remaining portion of segment IV), and 0.65 GBq (17.5 mCi) was delivered through the left hepatic artery (Figures 3(d) and 3(e)). A PET-CT scan 45 days later showed only a slight heterogeneous distribution of 18-FDG in the region of the previously observed lesions (Figures 1(c) and 1(f)), and the maximum standardized uptake value had declined to 4.2 from the pretreatment value of 8.5. A new abdominal MRI performed on the same day showed that the nodules had shrunk in both the right and left lobes (Table 2) [19]. All hepatic nodules were reduced in size by at least 50%, and 3 nodules disappeared, partial response according to mRECIST criteria.

After both SIRT treatments, the patient presented with Grade 1 fatigue that lasted approximately 1 week. Measurement of liver enzymes indicated that no liver damage had occurred (data not shown). Serum lipase concentrations progressively decreased from a high of 12,705 U/L to 344 U/L (Table 1), indicating control of the disease.

**Figure 4:**
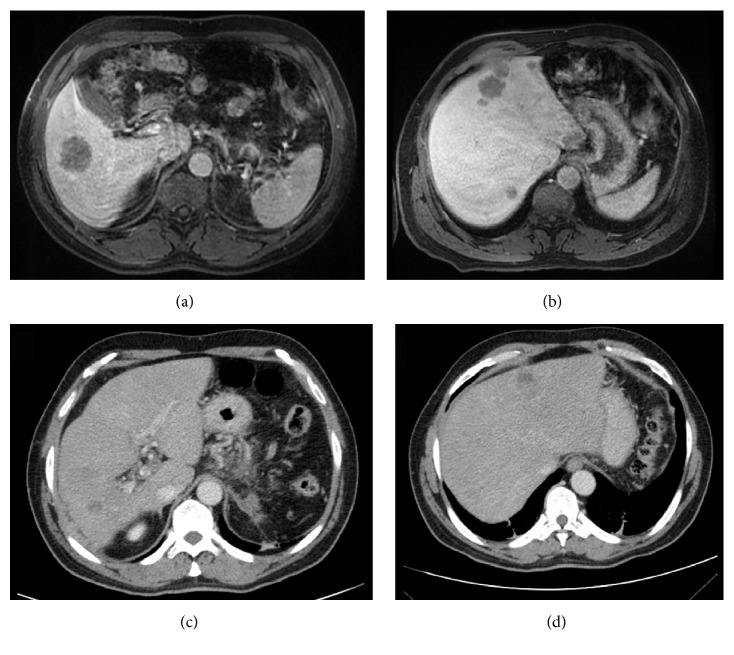
Comparison of baseline magnetic resonance imaging (MRI) (August 1, 2015) with computed tomography (CT) imaging performed on December 16, 2016. (a, b) Axial MRI images, portal phase, showing the hepatic nodules in the right (a) and left (b) lobes at admission. (c, d) Axial CT images, portal phase, showing regression of the hepatic nodules in the right (c) and left (d) lobes 15 months after the second SIRT and after several lines of chemotherapy.

The patient returned to the United States, where he has been under the care of an oncologist at the M. D. Anderson Cancer Center in Houston, Texas. Beginning 6 weeks after the second SIRT (November 3, 2015), he was treated with folinic acid-fluorouracil-oxaliplatin (FOLFOX) chemotherapy every 2 weeks for 6 cycles. Imaging on April 7, 2016, showed improvement in the hepatic lesions that did not reach the criteria for a partial response, and the FOLFOX treatment was continued. In July 2016, the hepatic lesions progressed; FOLFOX was discontinued, and chemotherapy with capecitabine was initiated. An abdominal MRI 82 days later showed hepatic lesions of 3.3 cm, 1.2 cm, and 1.0 cm in segment II and a 1.5 cm lesion in segment VII. The volume of the right hepatic lobe had decreased from April 7, and multiple millimeter-sized subcapsular and peritoneal lesions were evident. Chemotherapy with capecitabine was continued. Three weeks later, PET-CT confirmed the hepatic lesions and further indicated peritoneal carcinomatosis and involvement of the abdominal and retroperitoneal lymph nodes. A few days later, a CT-guided biopsy was performed, after which the chemotherapy regimen was changed to chronomodulated bevacizumab plus irinotecan with fluorouracil and folinic acid (FOLFIRI). To date, 2 cycles of bevacizumab plus FOLFIRI have been administered. CT images obtained on December 16, 2016 (490 days after the initial SIRT), indicated that the tumors had shrunk considerably (Figure 4). The patient is still alive today.

## 3. Discussion

Pancreatic acinar cell carcinoma is a rare neoplasm, representing 1% to 2% of exocrine pancreatic neoplasms in adults [2]. It occurs more frequently in men, with a peak incidence in the seventh decade of life [2, 3, 20]. Although survival is typically longer with PACC (median 18 to 30 months; 17 months for metastatic disease treated with chemotherapy) than it is with pancreatic ductal adenocarcinoma (a median of 6 months) [3, 20], metastasis is common; indeed, metastases are already present in half of patients at diagnosis, and recurrence has been reported in up to 72% of patients [3, 4].

In PACC, the tumor cells bear a morphological resemblance to acinar cells and express pancreatic enzymes such as trypsin, lipase, chymotrypsin, and amylase [1, 2]. The most common symptoms are abdominal pain and bloating, and 10% to 15% of patients develop lipase hypersecretion syndrome, which elevates blood lipase concentrations and can produce symptoms such as subcutaneous nodules resulting from fat necrosis and polyarthralgia from sclerotic lesions in cancellous bone [1, 2]. In our case, the patient had abdominal pain, and a diagnostic ultrasound found hepatic nodules of unknown origin. The patient also presented with a high serum lipase concentration of 8407 U/L (Table 1), despite not having clinical symptoms of lipase hypersecretion syndrome.

Surgical resection is associated with longer survival and is therefore always recommended when feasible, although it is rarely curative [3]. In our case, surgical resection of the primary tumor was possible despite the metastatic disease, and the patient underwent partial pancreatectomy (body plus tail). There are no clear recommendations for treating advanced PACC (metastases or locally unresectable tumors). Chemotherapy and radiotherapy have been used, but their effectiveness has been evaluated in only a small number of patients, with mixed results [2–4].

SIRT with Y-90 resin microspheres is a relatively new treatment modality that has been effective in treating liver metastases from several locations, such as the cervix, breast, lung, colon, rectum, and pancreas [7–10]. In 19 patients with liver metastases from pancreatic cancer, SIRT, used as salvage therapy, was associated with an objective response in the liver of 47% and a median overall survival of 9 months after treatment [11]. SIRT combined with systemic chemotherapy demonstrated 31% of partial response and 38% stable disease in the liver assessed using mRECIST; the median overall survival was 12.5 months after SIRT [13]. Fidelman and colleagues in a series of 3 cases of pancreatic tumors treated with SIRT verified that a patient with solid and papillary epithelial neoplasm (SAPEN) of the pancreas with hypervascular hepatic lesions at angiography got partial response assessed using mRECIST. On the other hand, patient with pancreatic adenocarcinoma did not respond to SIRT [15]. Recently, Michl et al. evaluated (pancreatic adenocarcinoma liver metastases treated with SIRT) tumor response according to PET Response Criteria In Solid Tumors (PERCIST). They found 35% of complete response, 6% of partial response, and 59% of disease progression. And the median overall survival was 8.8 months [21]. However, none of these patients had PACC.

In our patient, imaging showed multiple hypovascular liver nodules in both lobes, with no extrahepatic metastases, suggesting that he would be a good candidate for SIRT to attempt control of the liver disease. Follow-up imaging after SIRT revealed that the treatment was effective: all hepatic nodules were reduced in size by at least 50%, and 3 nodules disappeared (partial response according to mRECIST criteria).

Lipase hypersecretion syndrome occurs in a minority of patients with PACC, often in association with hepatic metastases, but can resolve with treatment [2]. Our patient's serum lipase concentrations decreased markedly after treatment with SIRT (Table 1). Considering that the lipase concentrations rose rather than fell after partial pancreatectomy, we attribute this finding to the efficacy of SIRT with Y-90 resin microspheres in reducing the burden of the liver metastases.

Chemotherapy with FOLFOX initiated after the SIRT initially shrank the tumors further, but the patient's disease subsequently progressed. Capecitabine treatment did not reverse the progression; however, 2 cycles of chronomodulated bevacizumab plus FOLFIRI dramatically shrank the tumors again, as shown with CT imaging 16 months after the first SIRT treatment.

SIRT was successful in reducing the burden of hepatic metastases from PACC; however multiple lines of systemic chemotherapy were needed to sustain the improvement as to treat also the lymph nodes at the hepatic hilum.

## Figures and Tables

**Table 1:** Laboratory examinations of an asymptomatic 59-year-old man with pancreatic acinar cell carcinoma treated with selective internal radiation therapy with Y-90 resin microspheres.

Laboratory measures	Timing of measurement^*∗*^			
	Before pancreatectomy	After pancreatectomy	After first SIRT	After second SIRT
	08/01/2015	08/13/2015	09/15/2015	10/30/2015
Alpha-Fetoprotein, IU/mL (normal range 0.0 to 5.8 IU/mL)	7.1	5.8	11.5	10.3
CEA, ng/mL (normal range 0.52 to 8.90 ng/mL)	1.39	0.78	1.04	2.06
CA 19-9, U/mL (normal range 2.50 to 34.0 U/mL)	1.61	0.60	1.65	1.25
Lipases, U/L (normal range 31 to 186 U/Lg)	8407	12,705	6387	344

CEA, carcinoembryonic antigen; CA, carbohydrate antigen; SIRT, selective internal radiation therapy. ^*∗*^Pancreatectomy was performed on 8/03/2015, first SIRT was performed on 8/14/2015, and second SIRT was performed on 9/18/2015.

**Table 2:** Measurement of liver nodules on magnetic resonance images before and after selective internal radiation therapy (SIRT) with Y-90 resin microspheres.

Segment & nodules (N)	Nodule size, cm						Reduction in nodule diameter after both SIRT treatments, %
	08/01/2015			09/15/2015		10/30/2015	
*Right lobe*		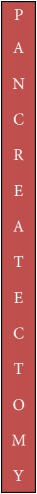	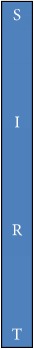				
*V*							
N1	5.3			3.0		2.2	58
*VI*							
N1	3.2			2.5		1.4	56
*VII*							
N1	1.1			0.4		0.0	100
N2	1.8			0.7		0.0	100
*Left lobe*					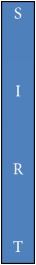		
*IV*							
N1	1.3			2.4		1.2	50
N2	2.5			4.2		1.8	57
N3	3.0			4.6		2.4	48
*II*							
N1	0.5			1.5		0.4	73
N2	0.8			1.2		0.0	100
N3	1.0			2.3		0.9	61
N4	2.7			4.5		2.0	55
